# Mechanical Stiffness Influences the Response of Human Uterine Fibroid Cells to Hormonal Treatments

**DOI:** 10.1007/s43032-025-02016-0

**Published:** 2025-12-08

**Authors:** Maria Victoria Bariani, Emnet Djibrila, Elise Maajid, Qiwei Yang, Ayman Al-Hendy, Mohamed Ali

**Affiliations:** 1 Department of Obstetrics and Gynecology, University of Chicago, Chicago, IL USA; 2 Rice University, Houston, TX USA; 3 Department of Medical Sciences, Khalifa University, Abu Dhabi, UAE; 4 Clinical Pharmacy Department, Faculty of Pharmacy, Ain Shams University, Cairo, Egypt

**Keywords:** Mechanical stiffness, Substrate rigidity, Leiomyoma cells, Steroid hormones, Hormone antagonist

## Abstract

Uterine fibroids (UFs), the most common gynecologic tumors in reproductive-age women, are characterized by the accumulation of extracellular matrix (ECM), which leads to increased stiffness. The stiffness can transfer mechanical signals from the ECM to the intracellular environment through a process called mechanotransduction, consequently altering the biological behavior of the cell. UFs are estrogen and progesterone-dependent, with hormonal fluctuations influencing their growth and development. Consequently, the emphasis in medical treatment development has been on options that target steroid hormones, including GnRH agonists/antagonists, aromatase inhibitors, and anti-progestins. However, there has been limited understanding of how mechanical stiffness influences the progression of UFs and their cells’ response to medical treatments. This work aimed to assess the impact of stiffness on UFs cell growth and on their responsiveness to hormone and anti-hormonal treatments. Our results showed that increased stiffness enhances proliferation and ECM accumulation in UF cells, and that the mechanical environment influences the response of these cells to both hormone and anti-hormone treatments. These findings offer valuable insights for understanding UF pathogenesis, racial disparity as well as improving UF treatment strategies, emphasizing the importance of considering the mechanical microenvironment in clinical approaches.

**Supplementary Information:**

The online version contains supplementary material available at 10.1007/s43032-025-02016-0.

## Introduction

Uterine fibroids (UFs, also known as leiomyoma or myoma) are the most common non-cancerous growths of the myometrium, affecting about 75% of reproductive-age women [1, 2]. While UFs are generally asymptomatic, they can manifest with clinical symptoms such as abnormal uterine bleeding, pelvic pain, and reproductive complications in up to 50% of afflicted women [3]. The exact etiology of UFs remains incompletely understood, involving a complex interaction of genetic, hormonal, and environmental factors [4, 5]. Notably, the foremost reported risk factor for UFs is race, with a disproportionate impact observed in both prevalence and severity among women of African ancestry [2, 6–10].

Although these benign tumors can vary in size and position within the uterus, a shared characteristic is the accumulation of extracellular matrix (ECM) [11, 12]. The ECM is the non-cellular component that surround the cells and plays key regulatory roles since it orchestrates cell signaling, functions, and morphology [13, 14]. The fibrous proteins and glycosaminoglycans (GAGs) that compose the ECM, along with their crosslinking, are responsible for its stiffness [15, 16]. This stiffness can transfer mechanical signals from the ECM to the intracellular environment through a process called mechanotransduction, consequently altering the biological behavior of the cell [16, 17]. Importantly, a fibrotic microenvironment is characterized by increased stiffness, and this rigidity is correlated with the progression of the disease [12].

UFs are estrogen and progesterone-dependent, with hormonal fluctuations influencing their growth and development [18, 19]. Consequently, the emphasis in medical treatment development has been on options that target steroid hormones, including GnRH agonists, aromatase inhibitors, and anti-progestins [20–22]. The GnRH antagonists Elagolix, Relugolix and, Linzagolix are the most recent approved oral treatments for the management of UF symptoms [21]. These antagonist in combination with hormonal replacement or add-back therapy have shown remarkable efficacy in controlling UF-related heavy menstrual bleeding [21, 23–25]. However, hysterectomy is the most chosen approach for managing UFs [26].

A recent study has demonstrated that UFs cells cultured on mechanically stiff substrates had enhanced progesterone receptor (PRB) activation [27], showing a potential connection between hormone and mechanical signaling pathways in UFs cells. However, there has been limited understanding of how mechanical stiffness influences the progression of UFs and their response to medical treatments. This work aimed to assess the impact of mechanical stiffness on UFs cell growth and their responsiveness to anti-hormonal treatments *in vitro*.

## Materials and Methods

### Human UFs Tissue Collection

Human UFs tissues were collected from self-reported Black or White premenopausal women undergoing hysterectomy for symptomatic UFs, following written informed patient consent under an IRB-approved protocol from the University of Chicago (IRB #20–1414). Patients self-reported their race as part of the demographic data collected upon admission. These patients had not received any hormonal supplements or treatment for 3 months prior to the day of surgery. UF primary cells were isolated from a freshly human fibroid sample collected from a self-identified Black premenopausal woman (41 years old). For nanoindentation measurement, patients were matched by age (Black 42.5 ± 1 vs. White 43.3 ± 1.5, p-value 0.6442) and BMI (Black 33.6 ± 1.1 vs. White 30 ± 3.7, p-value 0.3058). UFs tissues were flash-frozen and stored at −80 C until further use. Detailed clinical and demographic information for patients and fibroid samples, including age, BMI, gravidity/parity, menstrual cycle phase, and fibroid characteristics, is provided in Supplementary Table 1.

### Cell Culture

Immortalized human uterine leiomyoma (HuLM) cells (courtesy of Dr. Darlene Dixon) [28] were used for the study. HuLM cells (2 × 10^4^) were plated on Cytosoft® 6-well plates or T-25 flask of specific elastic modulus 0.2 kPa (Soft) and 64 kPa (Stiff) (Advanced BioMatrix; Carlsbad, CA) coated with PureCol® Type I collagen (100 µg/ml) (#5005, Advanced BioMatrix; Carlsbad, CA). HuLM cells were cultured in phenol-free DMEM/F-12, containing 10% heat inactivated fetal bovine serum and 1% Penicillin–Streptomycin antibiotics (#21041025, #A5256801, and #15140122 respectively, Thermo Fisher Scientific, Waltham, MA) at 37 °C in a humidified atmosphere of 5% CO2/95% air. After 24 h, HuLM cells were washed with PBS, trypsinized (TrypLE Express Enzyme, #12604021, Thermo Fisher Scientific, Waltham, MA), and centrifuged at 500 × g for 5 min. Supernatants were aspirated, and pellets were stored at −80 C until further use.

### Primary UF Cells Isolation

The UF tissue sample collected for primary cell isolation, was washed with calcium- and magnesium-containing Hanks’ balanced salt solution (HBSS, #14025076, Thermo Fisher Scientific, Waltham, MA) to remove blood, and chopped into small pieces. The tissue was digested for 3.5 h at 37 °C with shaking in an enzyme buffer of calcium- and magnesium-free HBSS containing 1% antibiotics-antimycotics, 2.5% N-2-hydroxyethylpiperazine-N′−2-ethanesulfonic acid (HEPES, #15630080, Thermo Fisher Scientific, Waltham, MA), 660 µg/mL collagenase Type IV (#NC9919937, Worthington, NJ, USA), and 4.76 µg/mL DNase I (#10104159001, Sigma-Aldrich, St. Louis, MO, USA). The suspension was filtered through a 100-µm sterile nylon mesh cell strainer to remove undigested tissues and then through a 70-µm cell strainer to obtain a single cell suspension (#CLS431752 and #CLS431751, respectively, Sigma-Aldrich, St. Louis, MO, USA). The remaining undigested tissue was suspended in a fresh enzyme buffer and incubated for 14 h at 37 °C and filtered again to obtain a single cell suspension. UF primary cells were plated out in regular T25 flask or 6-well plates (#FB012937 and #FB012927, Fisherbrand, Thermo Fisher Scientific, Waltham, MA) and incubated as described above.

### Cell Viability Assay

The CyQUANT™ XTT Cell Viability Assay (Catalog No. X12223, Thermo Fisher Scientific, Waltham, MA, US) was used to quantify the cellular metabolic activity as an indicator of cell viability and proliferation. One day before the experiment, the same number of HuLM cells or UF primary cells were seeded into CytoSoft 0.2 kPa (Soft) and 64 kPa (Stiff) 96-well plates and cultured overnight as specified above. The XXT colorimetric assay was assessed following manufactured instructions.

### Hormonal Agonists and Antagonist Treatments

HuLM cells were plated at a density of 10 × 10^3^ cells/cm^2^ in CytoSoft 0.2 kPa (Soft) 6-well plates and at a density of 15 × 10^3^ cells/cm^2^ in CytoSoft 64 kPa (Stiff) 6-well plates to account for slower growth in the soft plates. HuLM cells were cultured in previously stated conditions until they reach 70% confluence. Then, HuLM cells were washed thoroughly using PBS, and regular medium was replenished with phenol red-free DMEM/F-12 containing 5% charcoal stripped Fetal Bovine Serum (#12676029, Thermo Fisher Scientific, Waltham, MA) and 1% antibiotic). After HuLM were treated with estrogen (E2, 17β-Estradiol, #E2257, Sigma-Adrich, St. Louis, MO, US), progesterone (P4, Pregnene-3,20-dione, #P8783, Sigma-Adrich, St. Louis, MO, US) or the combination (E2 + P4) for 24 h. Then, ICI (E2 antagonist, #531042, Sigma-Adrich, St. Louis, MO, US) and Mifepristone (antagonist of P4 receptor, #S2606, Selleck Chemicals, Houston, TX, US) was added along with the corresponding hormone. Ethanol was used to dissolve E2 (10 nM) and P4 (40 nM) (final concentration < 0.1%). Dimethyl sulfoxide (DMSO, 472301, Sigma, St. Louis, MO, USA) was used to dissolve ICI (10 nM) and Mifepristone (10 nM) (final concentration < 0.1%). The same concentrations of ethanol and DMSO (< 0.1%) were used as a vehicle in the control cultures. After 24 h, HuLM cells were washed with PBS, trypsinized (TrypLE Express Enzyme, 12604021, Thermo Fisher Scientific, Waltham, MA), and centrifuged at 500 × g for 5 min. Supernatants were aspirated, and pellets were stored at −80 C until further use.

### RNA Isolation, cDNA Synthesis, and Quantitative Real-Time PCR

Total cellular RNA was isolated from frozen HuLM pellets using the RNeasy Kit (Catalog No. 74104, Qiagen, Hilden, Germany) following manufacturer instructions. RNA reverse transcription to complementary DNA (cDNA) was performed using Ecodry premix double-primed (#639549, Takara Bio, San Jose, CA, USA). Quantitative real-time PCR (qPCR) was carried out using SsoAdvanced Universal SYBR Green Supermix (Bio-Rad, Hercules, CA, USA) in a 10-μL final reaction volume (384 PCR plate). Primer sequences are listed in Supplementary Table 2. Primers were purchased from Integrated DNA Technologies (IDT, Coralville, IA, USA) except *ITGA5, ROCK1, and MYLK* that were purchased from Genecopoeia (Rockville, MD, USA). Real-time PCR analyses were performed using the Bio-Rad CFX384 detection system (Bio-Rad, Hercules, CA, USA). A melting-curve analysis affirmed the synthesis of a DNA product of the predicted size. The expression data were normalized using Gapdh RNA values, and these relative normalized values were used to generate data graphs. A reaction without a cDNA template was used as a negative control.

### Protein Expression Analysis by Western Blot

HuLM frozen cells pellets were lysed in RIPA buffer (#89900, Thermo Fisher Scientific, Waltham, MA) containing 1% of protease and phosphatase Inhibitor Cocktail (#78440, Thermo Fisher Scientific, Waltham, MA), vortexed, sonicated and centrifuged for 10 min at 12,000 RPM at 4◦C. Three experimental replicates per group were run. Samples equivalent to 20 µg of protein were separated using 4–20% Mini-PROTEAN TGX Precast Protein Gels (#4,561,096, Bio-Rad, Hercules, CA) and transferred to Trans-Blot Turbo Midi 0.2 µm PVDF membranes (#1704157, Bio-Rad, Hercules, CA) according to standard procedures. Membranes were blocked for 1 h at RT in either 5% w/v nonfat dry milk or 5% BSA in 0.1% Tween-supplemented PBS (0.1% PBS-T) per antibody specification. Membranes were then incubated with primary antibodies overnight at 4◦C in either 1% w/v nonfat dry milk or 1% BSA in 0.1% PBS-T per antibody specification. The following is the information regarding the primary antibodies used, including their source, and working dilutions: mouse anti-PCNA (ab29, Abcam; 1:1000), mouse anti-Cyclin D (ab16663, Abcam; 1:1000). Mouse anti-β-actin (A5441, Sigma, 1:10000) protein levels were assessed by re-probing the blots. Membranes were washed in 0.1% PBS-T and then incubated with anti-mouse (#7076, Cell Signaling; 1:5000) horseradish peroxidase-labeled antibodies. The antigen–antibody complex was detected with Trident femto Western HRP Substrate kit (GTX14698, GeneTex, Irvine, CA, USA) and images of immunoreactive bands were acquired using ChemiDoc XRS + molecular imager (Bio-Rad, Hercules, CA, USA). Bands were analyzed using Image J software [29]. The relative protein level was normalized to β-actin and results were expressed as relative optical density.

### Nanoindentation

To determine UFs tissue stiffness, we utilized the Piuma Nanoindenter (Optics11Life, Amsterdam, NE) instrument that was specially designed for the measurement of mechanical properties of complex and irregular materials, including tissues [30]. A 50 µm radius spherical probe with a cantilever stiffness of 0.5 N/m was used. For testing, a piece of frozen UF tissue was dissected at least 1 mm thick, balanced to room temperature, adhered to a 35 mm petri dish with Loctite® superglue, and covered with 1X PBS solution. At least 10–15 points were measured to address intra-sample variability. Tissues were indented to a fixed depth of 10 μm, and the probe’s position was held for 5 s. Notably, we chose to work with frozen samples to ensure all measurements were conducted under the same conditions and instrument calibration, minimizing variability. Multiple studies indicate that freeze–thaw cycles generally preserve key mechanical properties without adversely affecting tissue stiffness as compared to fresh tissues [31–34].

### Statistical Analysis

Comparisons between groups were made by two-tailed unpaired Student’s t-test using GraphPad Prism 9 (GraphPad Software, San Diego, CA). The assumption of normality was assessed by Shapiro–Wilks test. All data are presented as mean ± standard error of mean (S.E.M.). A difference between groups with *p < 0.05, **p < 0.005, ***p < 0.0005, or ****p < 0.0001 was considered statistically significant.

## Results

### Greater Mechanical Stiffness Increases Proliferation of UF Cells

**Fig. 1:**
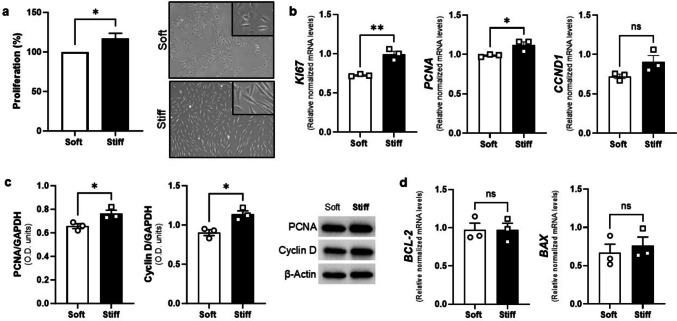
Stiffer substrate increases proliferation on HuLM cells. **a**) Percentage of cell viability (left) assessed using the XTT assay on HuLM cells cultured on CytoSoft® plates with different stiffness levels: Soft (0.2 kPa) and Stiff (64 kPa), and representative images captured using an inverted light microscope (right). Magnification = 4X Insets are enlarged image (10X) of the enclosed area. **b**) mRNA levels of the proliferation marker *KI67*, proliferating cell nuclear antigen (*PCNA*) and cyclin D (*CCND1*). **c**) Protein levels of proliferation markers PCNA and Cyclin D. **d**) mRNA levels of apoptosis markers B-cell lymphoma 2 (*BCL-2)* and BCL2 associated X (*BAX)*. Data are represented as mean ± SEM. ns = not significant. *p < 0.05, **p < 0.01

To investigate whether substrate stiffness influences proliferation and/or apoptosis in HuLM cells, we cultured them on soft or stiff plates (with elastic moduli of 0.2 kPa and 64 kPa, respectively) then explored proliferation/apoptosis using functional and molecular assays. Figure 1a shows that proliferation percentage is significantly greater in HuLM cultured in stiff as compared to soft plates. Additionally, we confirmed this observation on UF primary cells (Supplemental Fig. 1). To assess whether the increased proliferation phenotype observed in HuLM cell culture on a stiffer substrate correlates with molecular changes, we examined several proliferation markers at the gene and protein levels. We observed significantly elevated gene expression levels of *KI67* and *PCNA* (Fig. 1b) as well as the protein expression of the latter (Fig. 1c) in cells cultured on stiff plates compared to soft plates using qPCR and western blotting, respectively. As for cell cycle regulator marker Cyclin D, although we did not find statistically significant differences in *CDDN1* gene expression (Fig. 1a), we observed elevated Cyclin D protein levels in HuLM cells cultured on the stiffer substrate (Fig. 1b). Interestingly, when we assessed expression of two apoptosis related markers, antiapoptotic *BCL-2* and pro-apoptotic *BAX*, we did not observe significant differences in their mRNA levels between HuLM cells cultured on soft or stiff conditions. These results suggest that the enhanced cell viability illustrated in Fig. 1A is likely attributed to increased proliferation rather than a reduction in apoptosis.

### Stiffer Substrate Affects Extracellular Matrix Homeostasis and Mechanotransduction in UF Cells

**Fig. 2:**
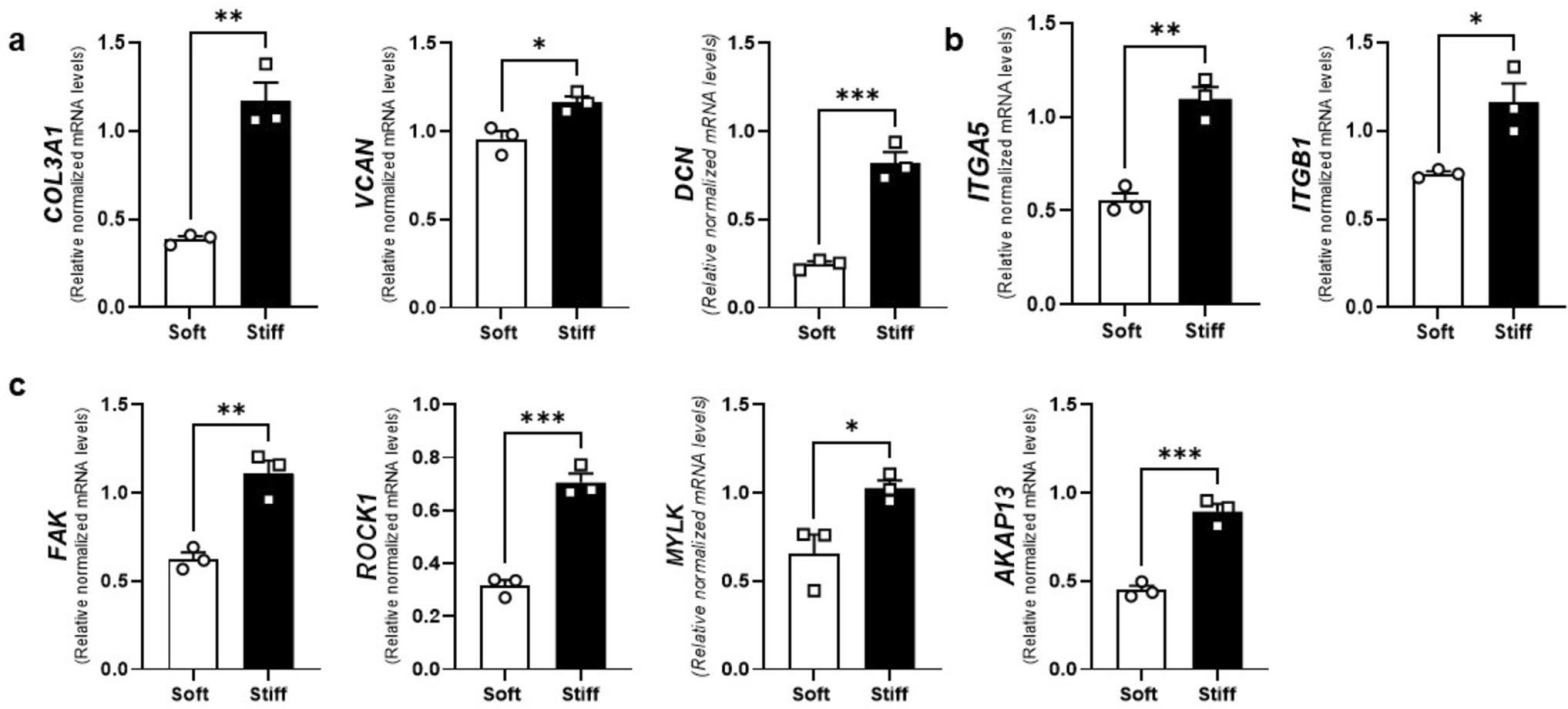
Stiffer substrate affects ECM and mechanosensing-related elements on HuLM cells. mRNA levels of **a**) the ECM elements collagen 3 alpha 1 chain (*Col3a1*), versican (*Vcan*), and decorin (*Dcn*), **b**) integrin alpha 5 (*Itga5)* and integrin beta 1(*Itgb1)* subunits, and **c**) mechanosensing elements focal adhesion kinase (*Fak*), rho-associated, coiled-coil-containing protein kinase 1 (*Rock1*), myosin light chain kinase (*Mylk*), and A-kinase anchoring protein 13 (*Akap13*) in HuLM cells cultured on Soft (0.2 kPa) and Stiff (64 kPa) plates. Data are represented as mean ± SEM. ns = not significant. *p < 0.05, **p < 0.01, ***p < 0.001

Next, we explored the impact of substrate rigidity on ECM accumulation and mechanotransduction-mediating elements expression. The mechanical characteristics of the ECM typically depend on three primary elements [35] fibrillar collagens such as *COL3A1* [36], glycosaminoglycans (GAGs) including Decorin (*DCN*) [37], and related proteoglycans, for example, Versican (*VCAN*) [38]. We observed statistically significant increases in the mRNA levels of these ECM constituents: *COL3A1*, *VCAN*, and *DCN* (Fig. 2a) in HULM cells cultured on stiffer condition as compared to soft condition. Moreover, since integrins are transmembrane heterodimeric receptors that sense the cell microenvironment and transduce biochemical signals into the cell and being particularly relevant in tumor progression [39]. Therefore, we evaluated the mRNA levels of α5β1 integrin, also called the fibronectin receptor [40]. We found increased mRNA levels of both *ITGA5* and *ITGB1* integrin subunits (Fig. 2b) in HULM cells cultured on stiffer condition. Additionally, we observed higher mRNA levels of several genes that are responsible for encoding kinases involved in mechanosensing pathways, including focal adhesion kinase (*FAK),* rho-associated, coiled-coil-containing protein kinase 1 *(ROCK1),* myosin light chain kinase (*MYLK* or *MCLK*)*,* and A-kinase anchoring protein 13 *(AKAP13)* along with stiffer culture condition (Fig. 2c). Collectively, these results indicate that ECM accumulation and mechanotransduction pathways are stimulated by the stiffer substrate.

### Substrate Stiffness Influence the Hormone-Induced Proliferation of UF Cells

**Fig. 3:**
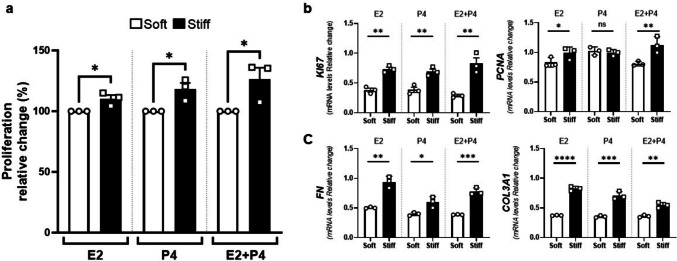
Stiffness substrate influences the hormones effect on HuLM cells. **a**) Cell viability was assessed using the XTT assay on HuLM cells treated with estrogen (E2, 10 nM), progesterone (P4, 40 nM) or the combination (E2 + P4) for 24 h cultured on CytoSoft® plates with different stiffness levels: soft (0.2 kPa, white bars) and stiff (64 kPa, black bars). The proliferation relative changes (%) in stiff relative to soft are shown as the mean ± SEM. mRNA levels of the **b**) proliferation markers, *Ki67* and *Pcna*, and **c**) ECM-related genes, *Fn* and *Col3a1*, on HuLM cells that were cultured on soft and stiff plates after Estrogen (E2, 10 nM), Progesterone (P4, 40 nM), or combination (E2 + P4) treatments for 24 h. Data are represented as mean ± SEM. ns = not significant. * = *p* < 0.05, ** = *p* < 0.01, *** = *p* < 0.001, **** = *p* < 0.0001, Student’s t test (Soft vs. Stiff)

The steroid hormones estrogen and progesterone play a pivotal role in the regulation of UF development and growth [18, 41]. Moreover, reports have demonstrated an interplay between steroid hormone and mechanotransduction signaling pathways in UFs [27, 42]. Therefore, we evaluated whether HuLM cells, cultured on soft vs. stiff plates, would respond differently to Estrogen (E2, 17β-Estradiol) or/and progesterone (P4, Pregnene-3,20-dione) treatments. Initially, we evaluated the effect of two different concentrations of hormones, alone or in combination, on HuLM proliferation cultured in regular plates (Supplementary Fig. 2A). We observed an increase in the cells proliferation after the treatment with the higher concentration of P4 (40 nM) alone or in combination with E2 10 nM. Consequently, these specific hormone concentrations were employed for remaining experiments. Our findings showed that HuLM cells cultured on the stiffer substrate under hormonal treatments exhibited higher proliferation rate compared to those cultured on the softer one (Fig. 3a). Subsequently, we observed that the mRNA levels of the proliferation markers *Ki67* and *Pcna* were higher in HuLM cells cultured on stiff compared to soft plates after hormone treatment, except for *Pcna* mRNA levels, which did not show differences after P4 treatment alone (Fig. 3b). Similarly, the mRNA levels of ECM markers *FN* and *COL3A1* were higher in HuLM cells cultured in stiff in comparison to soft plates after hormones treatments (Fig. 3c). These interesting data highlight, for first time, that UF cells respond differently to mechanical stiffness under hormonal stimulation, both at molecular and phenotype levels, which can play a role in explaining UF pathogenesis.

### The Response to Anti-Hormonal Treatment of UF Cells Is Determined by the Substate Stiffness

**Fig. 4:**
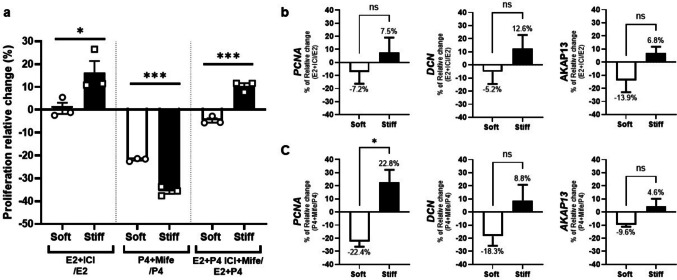
Stiffness substrate influences the hormones antagonist effect on HuLM cells. **a**) Percentage of cell viability relative change to the corresponding control, Estrogen (E2, 10 nM, left), Progesterone (P4, 40 nM, middle), or combination (E2 + P4, right) treatment assessed using the XTT assay. HuLM cells were cultured on CytoSoft® plates with different stiffness levels: Soft (0.2 kPa) and Stiff (64 kPa) and treated with ICI (left), P4 + Mifepristone (middle) and the antagonist combination (right). Percentage of *PCNA (left)*, Decorin (*DCN, middle*) and, A-kinase anchoring protein 13 (*AKAP13, right*) mRNA levels relative change to the corresponding control (hormones alone) after **b**) E2 + ICI and **c**) P4 + Mifepristone treatments in soft and stiff plates. After reaching 70–80% confluence, cells were treated with the corresponding treatment for 24 h. Data are represented as mean ± SEM. ns = not significant. T-student test (Soft vs. Stiff) *p < 0.05, ***p < 0.001

Next, we wanted to assess the impact of substrate stiffness on cells’ responses to anti-hormonal treatments. HuLM cells were treated with E2 antagonist ICI, antiprogestin Mifepristone, or their combination on both soft and stiff plates. Similar to hormonal treatment, we initially examined the effects of two different concentrations of each antagonist on HuLM proliferation when cultured in regular plates. We observed a decrease in cell proliferation following treatment with E2 and ICI (10 nM each), as well as P4 (40 nM) and Mifepristone (10 nM) (Supplementary Fig. 2b). Therefore, a concentration of 10 nM for the two antagonists was employed in the subsequent experiments. Interestingly, we observed that HuLM cells cultured on the soft substrate did not exhibit changes in proliferation, while those cultured on the stiff substrate continued to grow despite the ICI treatment (Fig. 4, left). Conversely, HuLM cells cultured in soft and stiff plates demonstrated a decrease in proliferation compared to the control (P4 alone) (Fig. 4, middle). Moreover, the decrease was significantly lower in HuLM cells cultured in stiff than in soft plates. Interestingly, after the combination of ICI and Mifepristone treatments, HuLM cells cultured in soft plates showed a decrease in proliferation relative to control (E2 + P4) whereas those cultured in stiff plates presented an increased proliferation relative to the control. Overall, these results demonstrate that the stiffness of the substrate determined the HuLM cells response to *in-vitro* anti-hormonal treatment.

### Uterine Fibroid Tissues from Black and White Patients Present Different Stiffness

**Fig. 5:**
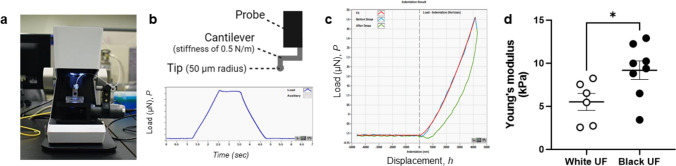
Nanoindentation of uterine fibroid tissues from Black and White women. **a**) Picture of the Piuma Nanoindenter (Optics11 Life, Amsterdam, Netherlands) that was used to test uterine fibroid tissues stiffness. **b**) Schematic of the spherical probe (50 μm radius, cantilever stiffness of 0.5 N/m) used for nanoindentation (top), and a representative load relaxation curve (bottom) generated from nanoindentation testing (samples were indented to a fixed depth of 10 μm for 5 s). **c**) Representative Load vs. Displacement indentation curve (Hertzian model). **d**) Quantification of Young’s modulus (tissue stiffness) in uterine fibroids from self-identified White (*n* = 6) and Black (*n* = 8) patients. Each point represents the average of 10–15 measurements in a uterine fibroid (medium size) from one patient. Data are represented as mean ± SEM. *p < 0.05. Student’s t-test. kPa: Kilopascal

A recent study demonstrated that Black women are more likely, than White women, to have UFs with increased tissue fibrosis and ECM accumulation. This suggests that ECM related molecular etiology can be contributing to the observed racial disparity in UFs. In the current work, we assessed the stiffness and strength measurements of UFs tissues collected from both Black and White women using the Piuma Nanoindenter and a spherical probe (Fig. 5a and b). We applied the Hertzian model as it is the preferred method for yielding reliable results regarding the *ex-vivo* tissue stiffness. We observed elevated tissue stiffness in UFs tissues from Black compared to White women (Fig. 5d). These data confirm the previous findings using more sensitive and advanced measurement and highlight that stiffness can, at least partly, contribute to UFs health disparity.

## Discussion

Uterine fibroids (UFs, or leiomyomas) are the most common benign gynecological tumors in up to 80% of premenopausal women [1, 43]. UF prevalence increases with age, and the highest prevalence is in women in their 40 s [44, 45]. Although benign, 25–50% of women with UFs experience significant morbidities that decrease health-related quality of life, and 30% report symptoms severe enough to miss work [46–48] including heavy and prolonged menstrual bleeding, pelvic pain, pressure, bulk symptoms, and infertility [10, 18, 49–51].. The total annual cost of UFs in the United States ranges from $5.9 billion to $42.2 billion, depending on the inclusion of direct and indirect costs, as well as evolving treatment options [52]. UFs are fibrotic tumors that are believed to be the product of driver mutation emergence in a perturbed myometrial stem cell (MMSC) followed by transformation into tumor-initiating SC (UFSC) which in turn seeds and maintains clonal tumor growth. This tumor growth is characterized by abundant ECM production (including collagen; COL, fibronectin; FN, proteoglycan, and laminins) that contributes to the bulk of these neoplasms [2, 53–59].

At a molecular level, this ECM network is not only crosslinked in a manner that supports cells and tissues, but also serves as a reservoir of bioactive molecules and growth factors. In addition, ECM can elicit cellular responses that are required for tissue morphogenesis, differentiation, and homeostasis [13] as well as physical cues that are converted into biochemical signals, resulting in intracellular biological changes in a process named mechanotransduction [60]. Therefore, aberrant ECM dynamics could lead to deregulated cell behaviors, resulting in pathological processes, including tissue fibrosis [61, 62]. In addition, the resulting altered mechanotransduction creates a pathological loop leading to altering gene expression in tumor cells and further increasing ECM production and thus tumor stiffness [11, 12, 16, 63, 64]. Current study has shown that UF cells exhibited higher proliferation accompanied with elevated expression of proliferation and cell cycle regulator molecular markers when grown in stiffer condition confirming the interplay between mechanical stiffness and biochemical cell signaling. Moreover, stiff culture condition resulted in increased expression of several ECM related markers including COL3A1, *DCN and VCAN* which represented main ECM elements. These data highlight the pathologic loop of increased ECM production in response to mechanotransduction which leads to further cell proliferation.

Notably, the genetic drivers dominantly responsible for stem cell transformation have been identified. Among these, somatic mutations in the gene encoding the RNA polymerase II transcriptional Mediator subunit 12 (MED12) are the most prevalent, accounting for ~ 70–80% of UFs [65–71]. Recent studies have linked *MED12* mutation with ECM accumulation, and our group previously showed that lentivirus mediated *MED12* silencing in UF cell line resulted in downregulation of several ECM related markers such as COL1A1 and FN along with suppressing TGFβ signaling [72]. More recent studies utilized CRISPR to introduce *MED12* mutation in differentiated myometrial cell lines with subsequent higher expression of several Collagens *in vitro* and more ECM accumulation *in vivo* using kidney capsule [73, 74]. Moreover, other recent studies explored differential expression of MED12-associated genes by comparing *MED12* mutant and wildtype UF tissues using RNA-sequencing; most of the aberrantly expressed genes were predominantly involved in the regulation of extracellular constituents [75]. While mechanical signals are agreed to play a central role in shaping cell and tissue function [60], several signaling pathways involved in UF pathogenesis have been shown to be activated by mechanical stretch [16, 27, 60]. Mechanical stretch was able to activate many kinases that regulate gene transcription, translation, cell proliferation and apoptosis, cellular senescence, and ECM metabolism [60]. Focal adhesion kinase (Fak) is one of these kinases that acts as an early mediator of integrin-mediated signaling that regulate cell survival and proliferation [76] since integrins receptors sense the cell microenvironment and transduce biochemical signals into the cell. In the current study, UF cells, under stiffer culture condition, expressed higher gene expression of *ITGA5* and *ITGB1* integrin subunits as well as several kinases such as *FAK, ROCK1, AKAP13* and *MYLK *highlighting the role stiffness can play on activating ECM and mechanosensing-related elements with subsequent tumor fibrosis.

Fibroids are hormone-dependent tumors where steroidal hormones, estrogen and progesterone, play crucial roles in the UF pathogenesis through promoting cell proliferation and enhancing growth factors production, such as transforming growth factor-β (TGF-β), that contribute to fibroid growth which create a favorable environment for the tumor growth [2, 41]. While our current study did not assess TGF-β expression in HuLM cells cultured on stiff plates, we recognize its critical role in UF pathogenesis and plan to investigate this in future experiments. Based on existing literature, we anticipate that TGF-β signaling will be more active in a stiffer microenvironment, as increased matrix stiffness has been shown to enhance TGF-β activation and downstream fibrotic signaling in various cell types. Studies have demonstrated that mechanotransduction can regulate TGF-β signaling by promoting the release of latent TGF-β and activating key downstream effectors such as SMADs [77–79]. We aim to explore this in future work to further elucidate the link between stiffness and fibrotic signaling in UF.

Moreover, these steroidal hormones have shown to play a central role in regulating the growth of these tumors and the accumulation of ECM through genomic and several nongenomic signaling pathways, including MAPK, ROCK, PI3K/AKT, and Smads [80]. Recent study showed that substrate stiffness on which fibroid cells are cultured modulated their responsiveness to progesterone receptor β (PRB) activation through MEK1/2 and Rho-ROCK kinase signaling dependent pathways [27]. Our current study revealed the role substrate stiffness can play on modulating fibroid cells’ proliferation in response to hormonal treatments, both at molecular level and phenotype level. In the current study, we confirmed our findings on primary cells isolated from patient tumor which are more physiologically relevant than cell line.

UFs are a major health disparity issue with a 3–4 times higher prevalence, regardless of age and symptoms status, in Black compared to White women [6–9, 44, 81–85]. This disproportionate disease burden on Black women adversely impacts their social competitiveness and well-being [86–90]. Our group and others have demonstrated, at the molecular level, distinct genetic, transcriptomic and epigenomic profiles in UF and myometrium tissues from Black vs. White women [91–99], suggesting a molecular basis for this health disparity with our two most recent studies highlighting role of ECM alteration and tissue fibrosis in this racial disparity [98, 99]. we employed a pilot study to compare the difference of stiffness between UFs from Black and White women using the Piuma Nanoindenter instrument that was specially designed for the measurement of mechanical properties of complex and irregular materials, including tissues [30]. Notably, using this approach, we observed a significant higher stiffness in UFs from Black in comparison to White women. These findings might provide new insights into molecular alterations correlating with racial disparities in UFs and improve our understanding of the molecular etiology underlying its pathogenesis. Interestingly, our results have shown that cells responded differently to not only hormonal treatments E2/P4 but also antihormonal ones including ICI (ER antagonist) or Mifepristone (PR antagonist) based on stiffness level. Uterine fibroid cells were more resistant to both antagonists when cultured on stiff culture condition compared to soft one in term of expected inhibition of cell proliferation as well as change of ECM and proliferation related markers levels. This novel data might provide basis to explain differential response to anti-UFs hormonal treatment among different racial population [100–102]. The heterogeneity of ECM protein types and quantities in relation to ethnicity and *MED12* mutation within individual UFs may contribute to varied treatment responses and need additional investigation. Few studies showed that UF ECM/stiffness can significantly affect its likelihood to respond to medical therapy [103, 104]. Moreover, a recent study revealed that patients with *MED12* exon 2 mutations had a significant smaller volume reduction after treatment with the GnRH agonist than those with UFs expressing wild-type *MED12* highlighting that *MED12* mutation status can predict the effect of hormonal treatment on UF reduction [105]. While our experiments did not directly investigate MED12 mutation status or its link to the ECM, prior studies have established a connection between MED12 mutations and ECM dysregulation in UF pathogenesis [69, 73]. Given that MED12 mutations are the most common genetic driver of UFs, this relationship supports the role of ECM in fibroid development. Additionally, study has shown that MED12 mutations influence the response to hormonal treatment [105]. This may help explain the differential treatment responses observed between Black and White patients, as prior research indicates that MED12 mutations are more prevalent in Black individuals. Our current and previous findings further suggest that UFs in Black patients exhibit increased stiffness, which may also contribute to these disparities [98, 99].

On the other hand, since cells possess the ability to sense the stiffness in the surrounding ECM and respond accordingly [106], recent study showed that growing UF cell line on different substrate stiffness can modulate their responsiveness to progesterone receptor (PR) activation, with greater stiffness induces greater PR activation [27], emphasizing the role stiffness can play in modulating cells response to hormonal exposure and thus tumor growth. Also, other study has shown that UF cells respond to manipulation of the mechanosensing Rho pathway differently when grown in collagen 1 Matrix 3D culture compared to 2D culture [107]. Collectively, it is imperative to better understand the correlations of UFs stiffness, clinical presentation, and treatment outcomes so that individualized approaches can be taken to optimize patient care. Moreover, the ability to predict UF response or lack of response to medical therapies is essential for such precision medicine planning for women with symptomatic UFs. Our results provide novel insights into the mechanical properties of UFs and their potential role in disease progression. Understanding the interplay between ECM stiffness and cellular behavior could open new avenues for targeted therapies, particularly those aimed at modifying the fibrotic environment. Given the established link between mechanical cues and fibroid pathophysiology, our findings may contribute to the development of treatment strategies that address both biochemical and biomechanical aspects of UFs. In Conclusion, our study showed that mechanical stiffness can affect fibroid cells resulting in increased proliferation and ECM production as well as modulate cell response to hormonal and antihormonal treatments. This can provide new insights into the role of mechanical forces, not only in UFs growth, but also on their response to pharmacological treatments. However, further analyses are necessary to better understand the underlying mechanisms.

## Supplementary Information

Below is the link to the electronic supplementary material.

Supplementary file1 (DOCX 120 KB)

## Data Availability

Included in manuscript.
