# Supplementary material for: Mechanical Stiffness Influences the Response of Human Uterine Fibroid Cells to Hormonal Treatments
Source: Reprod Sci. 2025 Dec 8;33(1):129–40. doi: 10.1007/s43032-025-02016-0 (PMC12948898; doi:10.1007/s43032-025-02016-0)
Supplement: Supplementary file 1 — Supplementary file1 (DOCX 120 KB) [file 43032_2025_2016_MOESM1_ESM.docx]

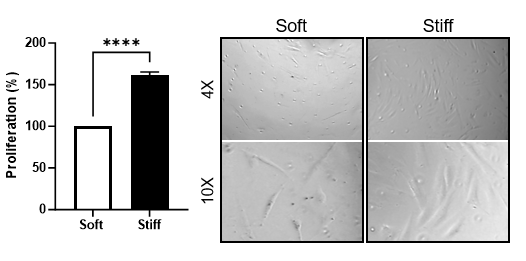


**Supplementary Figure 1. Stiffer substrate increases proliferation on primary UF cells.** Percentage of cell viability (left) assessed using the XTT assay on UF primary cells cultured on CytoSoft® plates with different stiffness levels: Soft (0.2 kPa) and Stiff (64 kPa), and representative images captured using an inverted light microscope (right).


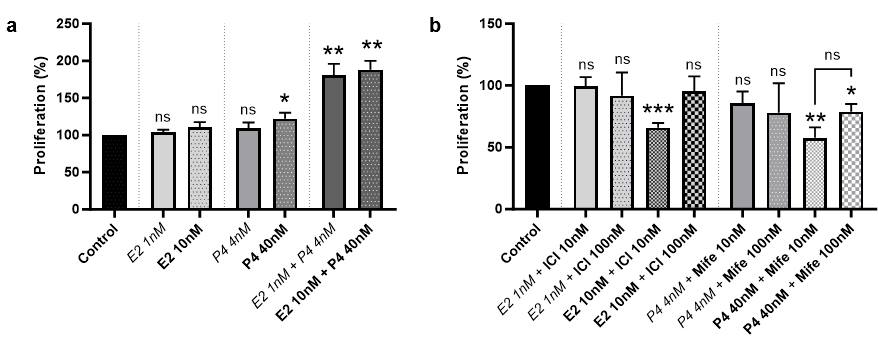


**Supplementary Figure 2.** **Effect of two different concentrations of hormones and antagonists on HuLM proliferation.** Percentage of cell viability assessed using the XTT assay on HuLM cells after 24 hours of treatment with two different concentrations of **a)** estrogen (E2, 17β-Estradiol), progesterone (P4, Pregnene-3,20-dione), or the combination (E2 + P4) or **b)** ICI (E2 antagonist), Mifepristone (antagonist of P4 receptor) along with the corresponding hormone. Statistical significance versus control denoted as *p<0.05, **p<0.01, ***p<0.001. ns= not significant.

**Table 1**: patients’ demographic information

| **Patient ID** | **Tissue** | **Gravida/Parity** | **Age** | **Race** | **BMI** | **Menstrual cycle phase** |
| --- | --- | --- | --- | --- | --- | --- |
| W#1 | Fibroid | G0P0 | 38 | White | 44.08 | Inactive endometrium |
| W#2 | Fibroid | G1P1 | 40 | White | 36.49 | Secretory endometrium. |
| W#3 | Fibroid | G4P3 | 46 | White | 25.42 | Interval endometrium. |
| W#4 | Fibroid | G4P3 | 46 | White | 23.82 | Secretory endometrium. |
| W#5 | Fibroid | G4P2 | 43 | White | 19.61 | Secretory endometrium. |
| W#6 | Fibroid | G1P0 | 47 | White | 30.27 | Inactive endometrium. |
| B#1 | Fibroid | G3P1 | 41 | Black/African American | 31.36 | Secretory endometrium. |
| B#2 | Fibroid | G4P3 | 41 | Black/African American | 39.13 | Inactive to weakly proliferative endometrium. |
| B#3 | Fibroid | G0P0 | 44 | Black/African American | 28.56 | Secretory endometrium. |
| B#4 | Fibroid | G3P3 | 48 | Black/African American | 35.09 | Inactive endometrium. |
| B#5 | Fibroid | G5P3 | 41 | Black/African American | 32.42 | Secretory endometrium. |
| B#6 | Fibroid | G1P1 | 45 | Black/African American | 34.95 | Secretory endometrium. |
| B#7 | Fibroid | G5P3 | 41 | Black/African American | 32.42 | Secretory endometrium. |
| B#8 | Fibroid | G3P3 | 39 | Black/African American | 34.79 | Secretory endometrium |

**Table 2.** Human primer sequences for RT-qPCR.

| **Symbol/Alias** | **Gene** | **Forward primer**  **sequence (5’-3’)** | **Reverse primer**  **sequence (5’-3’)** | |
| --- | --- | --- | --- | --- |
| *KI67* | Marker Of Proliferation Ki-67 | GAAAGAGTGGCAACCTGCCTTC | GCACCAAGTTTTACTACATCTGCC | |
| *PCNA* | Proliferating cell nuclear antigen | GCAAGTGGAGAACTTGGAAATG | GCCTAAGATCCTTCTTCATCCTC | |
| *CCND1* | *Cyclin D* | GTCAGCTCGTTACTCAACTC | GCTAGGCTTCCTGGTTTC | |
| *BCL-2* | B-cell lymphoma 2 | TTGTGGCCTTCTTTGAGTTCGGTG | GTGCCGGTTCAGGTACTCAGTCA | |
| *BAX* | BCL2 associated X | TCAGGATGCGTCCACCAAGAAG | TGTGTCCACGGCGGCAATCATC | |
| *COL3A1* | Collagen Type III Alpha 1 Chain | GCTGGCTACTTCTCGCTCTG | TCCGCATAGGACTGACCAAG | |
| *VCAN* | Versican | GAAAAGTCAGCCTACCTTATC | GATGCGGAGAAATTCACTGG | |
| *DCN* | Decorin | TGCTGTTGACAATGGCTCTC | GCCTTTTTGGTGTTGTGTCC | |
| *ITGA5* | Integrin Subunit Alpha 5 | Product ID: HQP117778 | | |
| *ITGB1* | Integrin Subunit Beta 1 | CGATGCCATCATGCAAGT | ACACCAGCAGCCGTGTAAC | |
| *AKAP13* | A-kinase anchoring protein 13 | CAGTGATGACATGGACAG | TCGGTGGATGAACTGGATC | |
| *ROCK1* | rho-associated, coiled-coil-containing protein kinase 1 | Product ID: HQP016371 | | |
| *FAK* | Focal adhesion kinase | CAGGGTCCGATTGGAAACCA | AAGCTTGACACCCTCGTTGT | |
| *MYLK* | Myosin light chain kinase | Product ID: HQP102705 | | |
| *GAPDH* | Glyceraldehyde-3-phosphate dehydrogenase | GTGGTCTCCTCTGACTTCAAC | | CCTGTTGCTGTAGCCAAATTC |
